# Prevalence of cognitive morbidity including delirium in 51,202 emergency hospital admissions across 29 medical and surgical specialties in ORCHARD-EPR: a cross-sectional study

**DOI:** 10.1016/j.eclinm.2025.103641

**Published:** 2025-11-24

**Authors:** Emily Louise Boucher, Sarah Catherine Smith, Sudhir Singh, Sasha Shepperd, Sarah Tamsin Pendlebury

**Affiliations:** a Wolfson Centre for Prevention of Stroke and Dementia, Wolfson Building, Nuffield Department of Clinical Neurosciences, University of Oxford, UK; b Departments of Acute General (Internal) Medicine and Geratology, Oxford University Hospitals NHS Foundation Trust, UK; c Nuffield Department of Population Health, University of Oxford, UK; d NIHR Biomedical Research Centre, Oxford University Hospitals NHS Foundation Trust, UK

**Keywords:** Delirium, Dementia, Hospital, Specialty, Cognitive morbidity, Older patients

## Abstract

**Background:**

Older people account for a growing proportion of unplanned hospital admissions and many have complex conditions. However, there are few data on cognitive morbidity (delirium, dementia and low cognitive test score) by specialty to plan services and guide policy. We therefore determined the occurrence of cognitive morbidity hospital-wide in older hospital patients using electronic patient record (EPR) data.

**Methods:**

The Oxford and Reading Cognitive Comorbidity, Frailty and Ageing Research Database (ORCHARD-EPR) includes data on consecutive patients aged ≥70 years with length of stay of ≥1 day (1st January 2017–31st December 2019) admitted to four hospitals covering Oxfordshire, UK. ORCHARD-EPR includes information from a mandatory on-admission cognitive screen comprising the 10-point Abbreviated Mental Test (AMT), dementia history and documentation of delirium where delirium diagnosis is based on a holistic assessment incorporating the AMT, Confusion Assessment Method (CAM) and clinical notes. Delirium and dementia diagnosis from the cognitive screen was supplemented by discharge ICD-10 coding. Prevalence of cognitive morbidity was determined hospital-wide and then by specialty.

**Findings:**

Among 51,202 admissions (mean/SD age = 82/7 years), any cognitive morbidity was present in 18,225 (35.6%, 95% CI 35.2–36.0%): delirium occurred in 24.0% (n = 12,289, of which 14.3% (n = 7332) had delirium only and 9.7% (n = 4957) had delirium + dementia) dementia only in 8.7%, (n = 4450), AMTS <8 in 2.9% (n = 1486). The prevalence of cognitive morbidity was highest in geriatrics (44.5%; n = 134/301), general medicine (42.8%; n = 14,346/33,512), trauma/orthopaedics (36.4%; n = 1337/3673), palliative care (36.0%; n = 128/356), stroke (30.8%; n = 144/468), infectious disease (27.6%; n = 42/152), neurosurgery (22.9%; n = 161/702) and general surgery (21.5%; n = 822/3819) and was 10–20% in all other specialties except two. Delirium was the most prevalent cognitive morbidity subtype in 24/29 specialties.

**Interpretation:**

Cognitive morbidity was common in older people with unplanned hospital admission across a broad range of specialties, with delirium accounting for most cases. Findings support the need for hospital-wide delirium screening and access to multidisciplinary team input for all specialties.

**Funding:**

Rhodes Trust, Canadian Institutes of Health Research, National Institutes for Health Research.

**Research in context**

**Evidence before this study**

We searched MEDLINE and EMBASE from inception to 20/01/2025 using key terms/words related to “Delirium”, “Dementia”, “Cognitive Impairment”, “Cognitive Defect/Decline” and “Hospitalisation”. We identified only four studies (mean age ∼80 years) reporting delirium (n = 2) or dementia (n = 2) across the entire acute hospital population with no study reporting overall cognitive morbidity. Delirium prevalence was 15% when ascertained using the 4 A's test (4AT) and Diagnostic and Statistical Manual of Mental Disorders-V criteria in a convenience sample (n = 1507) and 21% using retrospective chart review methods (n = 626) with dementia prevalence of 18% and 25% (n = 598 and 1439). Specialty-specific cognitive morbidity data were scarce with limited information only on broad specialty categories.

**Added value of this study**

We used data from the Oxford and Reading Cognitive Comorbidity, Frailty and Ageing Research Database-Electronic Patient Records (ORCHARD-EPR) to determine cognitive morbidity (delirium, dementia, low cognitive test score) hospital-wide and then across 29 acute specialties. ORCHARD-EPR contains detailed cognitive and delirium screening data on patients aged ≥70 years in whom screening is mandatory irrespective of specialty in our institution. In 51,202 admissions (2017–2019; mean/SD age 82/7 years, 73% screened), any cognitive morbidity was present in all 29 acute specialties examined with overall prevalence of 36%, range 10–45%. Prevalence was high as expected in general and geriatric medicine, and trauma and orthopaedics but also in a number of other specialities including palliative care, infectious diseases, general surgery, neurosurgery, oncology, ophthalmology, hepatology and vascular surgery. Delirium, alone or superimposed on dementia, was the most prevalent form of cognitive morbidity across all age groups and in 24/29 medical and surgical specialties with delirium alone being more frequent than delirium superimposed on dementia

**Implications of all the available evidence**

Cognitive morbidity was present in over one-third of patients aged 70 years or older across a broad range of acute hospital specialties including many not previously associated with the care of complex older patients. Most patients had delirium and did not have a dementia diagnosis. Our findings have implications for policy and for service design and delivery: cognitive and delirium screening for older patients should be implemented hospital-wide to identify those with cognitive morbidity supported by appropriate staff training and multidisciplinary team input to optimise care for this vulnerable patient group.

## Introduction

Older people account for a growing proportion of emergency hospital admissions globally.^1^ The majority are admitted to general medicine but other specialities are increasingly providing care to older patients. As many as a quarter to half of older hospital patients have moderate or severe (physical) frailty^2^ but less is known about the burden of the different subtypes of cognitive morbidity, ie delirium, dementia and low cognitive test score which are poorly captured by most frailty tools. Cognitive morbidity is an important determinant of short- and long-term care needs, mortality and functional decline but a lack of reliable hospital-wide estimates by age, sex and specialty limits service-planning, guidelines and policy for improving the outcomes of this population (Supplementary Box S1).^1^^,^^3^, ^4^, ^5^

Research in older acute hospital patients with cognitive morbidity is challenging for practical and ethical reasons and data are scarce (Supplement Literature Review, Tables S1 and S2). Hospital-wide delirium occurrence has been reported in two studies but both had limitations.^6^^,^^7^ One was a point prevalence study using a convenience sample where delirium was ascertained across the hospital in a single day and reported for a handful of specialties, the other used retrospective chart review. Although widely available across institutions internationally, administrative diagnostic International Classification of Diseases (ICD)-10 coding for delirium is underused and not always accurate.^8^^,^^9^ More reliable delirium ascertainment is possible in prospective cohorts but these are labour intensive and generally focussed on specific populations or settings. In general (internal) medicine, delirium affects around a quarter of adults and one third of those aged over 65 years but findings may not generalise to the hospital at large or to other acute specialties.^3^

Similarly, there are few studies on hospital-wide dementia occurrence. As with delirium, methods of ascertainment vary making comparisons across studies difficult. ICD-10 diagnostic coding for dementia in administrative datasets is more sensitive than for delirium but nevertheless many hospital patients have undiagnosed dementia^10^ and only around 60% of diagnosed dementia is coded.^9^ Two hospital-wide prospective studies using Diagnostic and Statistical Manual of Mental Disorders (DSM) criteria to diagnose dementia reported dementia prevalence of 18% at ≥65 years and 25% at ≥70 years overall with a higher occurrence in medical versus surgical specialties but detailed specialty level breakdowns were not provided.^11^^,^^12^

We determined the burden of cognitive morbidity hospital-wide and by specialty using the same methodology in all acute hospitals across a defined region using the dedicated Oxford and Reading Cognitive Comorbidity, Frailty and Ageing Research Database-Electronic Patient Records (ORCHARD-EPR).^13^ ORCHARD-EPR exploits individual Electronic Health Records (EHRs) acquired as part of standard care and importantly, includes the results of mandatory hospital-wide cognitive screening including delirium diagnosis based on a holistic assessment informed by the 10-point Abbreviated Mental Test (AMT), the Confusion Assessment Method (CAM),^14^ and documentation of pre-existing dementia diagnosis.^15^ We determined the prevalence of delirium, dementia and low cognitive test score overall and stratified by age, sex and acute care specialty in all patients aged 70 years or over admitted as an emergency.

## Methods

### Study design and participants

Oxford University Hospitals NHS Foundation Trust (OUHFT) consists of four general hospitals (John Radcliffe Hospital, Nuffield Orthopaedic Centre and Churchill Hospital in Oxford, and the Horton General Hospital in Banbury) providing all acute secondary care for all specialties in Oxfordshire, UK with a catchment area of >800,000 people. The Oxfordshire population is older than the England average, with lower average deprivation but all deprivation deciles are represented. According to the 2021 census, 86% of the population is white.

This was an observational study that used individual EHR data from ORCHARD-EPR^13^ including cognitive screening data collected as part of routine care. EHR data on all hospital patients aged ≥70 years with emergency admission were extracted by the hospital information analysts, pseudonymised and entered into the database for analysis by the research team. The study was reported according to STROBE guidelines.

We included all unplanned admissions regardless of specialty occurring between 1st January 2017 (when EHRs were fully implemented) and 31st December 2019 (prior to the onset of the Covid pandemic) with an inpatient length of stay ≥1 day. The treating specialty was identified using the treatment function variable and included specialties reporting n > 50 admissions over the study period. All other clinical data were obtained from the EHRs. Illness severity/acuity was measured using the National Early Warning Score (NEWS) version 1.^16^ Diagnostic ICD-10 codes were used to calculate the Charlson co-morbidity index (CCI),^17^ a composite measure of co-morbidity with updated weightings, and a modified Hospital Frailty Risk Score (HFRS), calculated from 109 frailty-related weighted ICD-10 codes, excluding coded delirium with moderate frailty defined as HFRS = 5–15 and severe frailty as HFRS >15 (Supplementary Methods).^2^^,^^18^

### Cognitive assessment

The electronic cognitive screen and its implementation via the EHR (Cerner Millenium) has been described previously (Supplementary Methods and Figure S2).^3^^,^^19^^,^^20^ Briefly, the screen included: the 10-point AMT^15^; delirium diagnosis made as part of a holistic assessment done as part of the clerking process and informed by the CAM (individual CAM items/the CAM score were not recorded), the AMT, notes review and informant interview where relevant.^14^ Delirium diagnosis was recorded in response to the question “Does the patient have delirium?” with answer yes, uncertain or no; and documentation of dementia by the question “Does the patient have a known diagnosis of dementia?”

The CAM was chosen as the recommended delirium screening tool in use at the time whereas the 4 A's test (4AT) is now the recommended screening test in the UK.^21^ The electronic cognitive proforma was designed to be completed even in patients who were untestable with the AMT. The proforma was triggered automatically on admission for all inpatients aged ≥70 years for completion usually by the resident doctor as an integral part of the clerking process (history and physical examination) but could also be completed ad hoc during admission.

Data from cognitive screening was supplemented with the ICD-10 diagnostic codes for delirium and dementia applied by trained medical coders after patient discharge according to standard OUHFT procedures (Supplementary Methods).^9^ ICD-10 codes were included to capture delirium and dementia recorded as free-text in the EHR where the cognitive screening proforma had not been completed or where delirium or dementia were recorded as absent in the proforma. “Certain” delirium was defined as delirium = yes on the screening proforma or presence of delirium ICD-10 code and “uncertain” delirium as delirium = uncertain and no ICD-10 delirium code. For the current study, delirium was defined to include both certain and uncertain delirium based on our previous work demonstrating that the combined certain and uncertain delirium prevalence approaches the expected delirium prevalence and that uncertain delirium has similar characteristics and outcomes to certain delirium.^19^^,^^20^

### Ethics

ORCHARD-EPR is approved by the National Health Service Health Research Authority (Oxford South Central Research Ethics Committee reference: 18/SC/0184). There is a waiver of individual consent although individuals may opt-out.

### Statistics

We determined prevalence of cognitive morbidity overall and for four mutually exclusive diagnostic groups: delirium alone (without pre-existing dementia diagnosis), delirium superimposed on dementia, dementia alone (without delirium) and low AMT score (<8) in the absence of a delirium or dementia diagnosis. We calculated prevalence by dividing the number of admissions with cognitive morbidity by the total number of admissions including those who did not receive a cognitive screen or ICD-10 code for delirium or dementia and determined 95% confidence intervals using Wilson's method for binomial proportions. Descriptive data were compared using the Kruskal–Wallis rank sum test (continuous data) and the Pearson's Chi-squared test (nominal data) as appropriate including in comparing clinical characteristics across cognitive morbidity subtypes (delirium, delirium superimposed on dementia, dementia and AMTS <8 without delirium or dementia diagnosis). Prevalence of cognitive morbidity by age group was evaluated using the Cochran–Armitage test for trend. Z-test was used as a post-hoc test for age-stratified male versus female comparisons within a given type of cognitive morbidity corrected using Bonferroni method for multiple comparisons. Analyses were done in R version 4.2.1.

### Role of the funding source

The funders had no role in the design and conduct of the study, interpretation of the data or decision to submit the manuscript for publication.

## Results

Of 51,202 included admissions (mean/SD age of 82/7 years; 51% female), over half had moderate (n = 23,683 [46.3%]) or severe (n = 5945 [11.6%]) physical frailty and 14,083 (27.5%) had an abnormal NEWS score (Table 1). A total of 33,512 (65%) of admissions were to general internal medicine (Table 2, Table S3). Of the remainder, 5613 (11%) admissions were to other specialist medical services, 3819 (7.5%) to general surgery, 3673 (7.2%) to trauma and orthopaedics and 4585 (9.0%) were to other specialist surgical services. Cognitive assessments, including a completed cognitive screen or ICD-10 codes documenting dementia or delirium diagnosis, were available for 37,478 (73%) of the cohort overall and 31,970 (78%) of general internal medicine, general surgery and trauma and orthopaedics admissions, which together accounted for 41,004 (80%) of all admissions. Missing cognitive assessments were associated with younger age and lower levels of comorbidity, frailty and illness severity (all *p* < 0.0001, Wilcoxon rank sum test/Pearson's Chi-squared test; Table S4).

**Table 1:** Clinical and demographic characteristics for the acute hospital-wide cohort overall and by cognitive morbidity and cognitive morbidity subtypes in ORCHARD-EPR 2017–2019.

Characteristic	Overall N = 51,202	Any cognitive morbidityN = 18,225	Delirium N = 7332	Delirium superimposed on dementia N = 4957	Dementia N = 4450	AMTS <8 without delirium or dementia diagnosis N = 1486	*p*-value^a^
Age, mean (SD)	82 (7)	85 (7)	84 (7)	86 (6)	85 (7)	85 (7)	<0.0001
Female^b^	25,858 (50.5)	9937 (54.5)	3899 (53.2)	2756 (55.6)	2458 (55.2)	824 (55.5)	0.016
Care home resident	7567 (14.8)	4710 (25.8)	1321 (18.0)	1798 (36.3)	1327 (29.8)	264 (17.8)	<0.0001
CCI, mean (SD)	11 (10)	15 (11)	10 (9)	21 (9)	20 (9)	9 (9)	<0.0001
HFRS, mean (SD)	7 (6)	11 (7)	9 (6)	14 (7)	11 (6)	8 (5)	<0.0001
No or mild frailty	21,574 (42.1%)	3353 (18.4%)	1716 (23.4%)	403 (8.1%)	768 (17.3%)	466 (58.7%)	<0.0001
Moderate frailty	23,683 (46.3%)	10,455 (57.4%)	4416 (60.2%)	2570 (51.8%)	2596 (58.3%)	873 (58.7%)	
Severe frailty	5945 (11.6%)	4417 (24.2%)	1200 (16.4%)	1984 (40.0%)	1086 (24.4%)	147 (9.9%)	
Abnormal NEWS^b^	14,083 (27.5)	5842 (32.4)	2523 (34.7)	1662 (33.9)	1272 (28.9)	385 (26.0)	<0.0001

Numbers are n (%) unless otherwise specified.

CCI, Charlson Comorbidity Index (a validated measure of comorbidity burden derived from ICD-10 codes); HFRS, modified Hospital Frailty Risk Score excluding coded delirium, a validated measure of frailty derived from ICD-10 codes; <5, mild or no frailty (low risk); 5–15 moderate frailty (intermediate risk), ≥15 severe frailty (high risk), NEWS, National Early Warning Score; SD, Standard Deviation.

a Kruskal–Wallis rank sum test (continuous data); Pearson's Chi-squared test (nominal data) were used to compare characteristics across types of cognitive morbidity (delirium, delirium superimposed on dementia, dementia and AMTS <8 without delirium or dementia diagnosis).

b Sex missing for n = 508; NEWS missing for n = 485.

**Table 2:** Specialty-specific prevalence of cognitive morbidity subtypes (specialties with n > 50; n = 50,950).

Specialty	Mean (SD) Age	Any cognitive morbidity N % (95% CI)	Delirium N % (95% CI)	Delirium on dementia N % (95% CI)	Dementia N % (95% CI)	AMTS <8 without delirium or dementia diagnosis N % (95% CI)
Geriatric Medicine N = 301	83.0 (6.9)	134/301 44.5% [38.8–50.3%]	63/301 20.9% [16.6–26.1%]	34/301 11.3% [8.06–15.6%]	28/301 9.30% [6.38–13.3%]	9/224 4.02% [1.97–7.74%]
General Internal Medicine N = 33,512	83.2 (7.3)	14,346/33,512 42.8% [42.3–43.3%]	5695/33,512 17.0% [16.6–17.4%]	4161/33,512 12.4% [12.1–12.8%]	3368/33,512 10.1% [9.73–10.4%]	1122/24,604 4.56% [4.30–4.83%]
Trauma and Orthopaedics N = 3673	82.6 (7.5)	1337/3673 36.4% [34.8–38.0%]	467/3673 12.7% [11.7–13.8%]	456/3673 12.4% [11.4–13.5%]	327/3673 8.90% [8.01–9.88%]	87/2802 3.10% [2.51–3.83%]
Palliative Medicine N = 356	79.4 (6.3)	128/356 36.0% [31.0–41.2%]	109/356 30.6% [25.9–35.7%]	5/356 1.40% [0.52–3.44%]	4/356 1.12% [0.36–3.05%]	10/210 4.76% [2.44–8.84%]
Stroke Medicine N = 468	82.1 (7.1)	144/468 30.8% [26.7–35.2%]	69/468 14.7% [11.7–18.4%]	29/468 6.20% [4.26–8.88%]	40/468 8.55% [6.25–11.5%]	6/183 3.28% [1.34–7.33%]
Infectious Diseases N = 152	82.1 (7.3)	42/152 27.6% [20.8–35.6%]	13/152 8.55% [4.82–14.5%]	11/152 7.24% [3.85–12.9%]	13/152 8.55% [4.82–14.5%]	5/102 4.90% [1.82–11.6%]
Neurosurgery N = 702	77.9 (6.0)	161/702 22.9% [19.9–26.3%]	116/702 16.5% [13.9–19.5%]	10/702 1.42% [0.73–2.69%]	31/702 4.42% [3.07–6.28%]	4/233 1.72% [0.55–4.63%]
General Surgery N = 3819	80.2 (6.9)	822/3819 21.5% [20.2–22.9%]	301/3819 7.88% [7.06–8.79%]	125/3819 3.27% [2.74–3.90%]	264/3819 6.91% [6.14–7.78%]	132/2868 4.60% [3.88–5.45%]
Ophthalmology N = 117	80.7 (7.1)	24/117 20.5% [13.8–29.2%]	2/117 1.71% [0.30–6.65%]	1/117 0.85% [0.04–5.36%]	14/117 12.0% [6.94–19.6%]	7/85 8.24% [3.66–16.8%]
Hepatology N = 67	77.5 (5.9)	13/67 19.4% [11.1–31.2%]	10/67 14.9% [7.77–26.2%]	0/67 0% [0–6.76%]	2/67 2.99% [0.52–11.3%]	1/38 2.63% [0.14–15.4%]
Vascular Surgery N = 773	80.4 (6.3)	141/773 18.2% [15.6–21.2%]	51/773 6.60% [5.00–8.64%]	15/773 1.94% [1.13–3.26%]	57/773 7.37% [5.68–9.51%]	18/423 4.26% [2.62–6.77%]
Gastroenterology N = 183	78.1 (6.2)	31/183 16.9% [12.0–23.3%]	16/183 8.74% [5.24–14.1%]	4/183 2.19% [0.70–5.86%]	9/183 4.92% [2.42–9.42%]	2/101 1.98% [0.34–7.66%]
Thoracic Surgery N = 86	78.9 (7.1)	14/86 16.3% [9.50–26.1%]	3/86 3.49% [0.91–10.6%]	4/86 4.65% [1.50–12.1%]	5/86 5.81% [2.16–13.7%]	2/35 5.71% [1.00–20.5%]
Spinal Surgery N = 200	78.2 (5.8)	32/200 16.0% [11.4–22.0%]	17/200 8.50% [5.18–13.5%]	1/200 0.50% [0.03–3.18%]	13/200 6.50% [3.65–11.1%]	1/101 0.99% [0.05–6.18%]
Clinical Oncology (Radiotherapy) N = 234	77.6 (5.4)	37/234 15.8% [11.5–21.3%]	26/234 11.1% [7.52–16.0%]	1/234 0.43% [0.02–2.73%]	4/234 1.71% [0.55–4.61%]	6/112 5.36% [2.20–11.8%]
Maxillo Facial Surgery N = 91	79.6 (7.0)	14/91 15.4% [8.97–24.8%]	4/91 4.40% [1.42–11.5%]	3/91 3.30% [0.86–10.0%]	5/91 5.49% [2.04–12.9%]	2/32 6.25% [1.09–22.2%]
Renal Medicine N = 311	78.3 (5.7)	45/311 14.5% [10.9–19.0%]	32/311 10.3% [7.25–14.3%]	4/311 1.29% [0.41–3.49%]	5/311 1.61% [0.59–3.93%]	4/178 2.25% [0.72–6.02%]
Urology N = 856	80.1 (6.7)	123/856 14.4% [12.1–16.9%]	28/856 3.27% [2.22–4.76%]	17/856 1.99% [1.20–3.23%]	61/856 7.13% [5.54–9.11%]	17/624 2.72% [1.65–4.42%]
Ear Nose and Throat N = 685	81.3 (7.0)	95/685 13.9% [11.4–16.7%]	25/685 3.65% [2.42–5.42%]	12/685 1.75% [0.95–3.13%]	55/685 8.03% [6.16–10.4%]	3/270 1.11% [0.29–3.48%]
Gynaecology N = 96	77.6 (7.0)	13/96 13.5% [7.69–22.4%]	4/96 4.17% [1.34–10.9%]	4/96 4.17% [1.34–10.9%]	5/96 5.21% [1.93–12.3%]	0/41 0% [0–10.7%]
Medical Oncology N = 853	76.3 (5.3)	112/853 13.1% [11.0–15.6%]	81/853 9.50% [7.65–11.7%]	9/853 1.06% [0.52–2.07%]	7/853 0.82% [0.36–1.76%]	15/405 3.70% [2.16–6.17%]
Plastic Surgery N = 386	80.5 (6.9)	50/386 13.0% [9.85–16.8%]	9/386 2.33% [1.14–4.54%]	7/386 1.81% [0.80–3.87%]	33/386 8.55% [6.04–11.9%]	1/135 0.74% [0.04–4.67%]
Clinical Haematology N = 708	77.6 (5.3)	87/708 12.3% [10.0–15.0%]	55/708 7.77% [5.95–10.1%]	6/708 0.85% [0.34–1.93%]	17/708 2.40% [1.45–3.90%]	9/310 2.90% [1.42–5.63%]
Respiratory Medicine N = 220	78.9 (6.1)	27/220 12.3% [8.38–17.5%]	14/220 6.36% [3.66–10.7%]	3/220 1.36% [0.35–4.26%]	7/220 3.18% [1.40–6.72%]	3/106 2.83% [0.73–8.65%]
Cardiac Surgery N = 254	77.5 (5.7)	29/254 11.4% [7.90–16.1%]	19/254 7.48% [4.68–11.6%]	3/254 1.18% [0.31–3.70%]	5/254 1.97% [0.73–4.79%]	2/101 1.98% [0.34–7.66%]
Neurology N = 141	77.6 (5.2)	15/141 10.6% [6.28–17.2%]	8/141 5.67% [2.66–11.2%]	2/141 1.42% [0.25–5.55%]	3/141 2.13% [0.55–6.57%]	2/74 2.70% [0.47–10.3%]
Cardiology N = 1462	79.5 (6.5)	149/1462 10.2% [8.71–11.9%]	64/1462 4.38% [3.41–5.59%]	17/1462 1.16% [0.70–1.90%]	57/1462 3.90% [2.99–5.06%]	11/478 2.30% [1.21–4.21%]
Upper Gastrointestinal Surgery N = 59	75.8 (5.0)	5/59 8.47% [3.16–19.4%]	3/59 5.08% [1.32–15.1%]	0/59 0% [0–7.62%]	1/59 1.69% [0.09–10.3%]	1/29 3.45% [0.18–19.6%]
Transplant Surgery Service N = 185	74.1 (4.0)	4/185 2.16% [0.69–5.80%]	4/185 2.16% [0.69–5.80%]	0/185 0% [0–2.54%]	0/185 0% [0–2.54%]	0/40 0% [0–10.9%]

At least one cognitive morbidity disorder was present in 18,225/51,202 (35.6%, 95% CI 35.2–36.0%) of admissions hospital-wide with no change over the three years of data collection (data not shown). Patients with cognitive morbidity were more likely to be older, resident in a care home, and to be comorbid, frail and severely ill (all p < 0.0001, Wilcoxon rank sum test/Pearson's Chi-squared test; Table 1, Table S5). Delirium was the most frequent diagnosis, occurring in 12,289 (24.0%, 95% CI 23.6–24.4%) of admissions overall. When stratified by dementia status, delirium alone occurred in 7332 (14.3%, 14.0–14.6%) of admissions and delirium superimposed on dementia in 4957 (9.7%, 9.4–9.9%), whereas dementia in the absence of delirium was present in 4450 (8.7%, 8.5–8.9%). Of patients with a completed cognitive screen, 27,451/34,164 (80.4%) had an AMT completed and 1486 (2.9%, 2.8–3.0% of the cohort overall) had deficits on cognitive testing (AMT<8) in the absence of delirium or dementia diagnosis. Restricting the analysis to certain delirium resulted in lower prevalence of cognitive morbidity overall but delirium remained the most frequent subtype (Table S6).

**Fig. 1:**
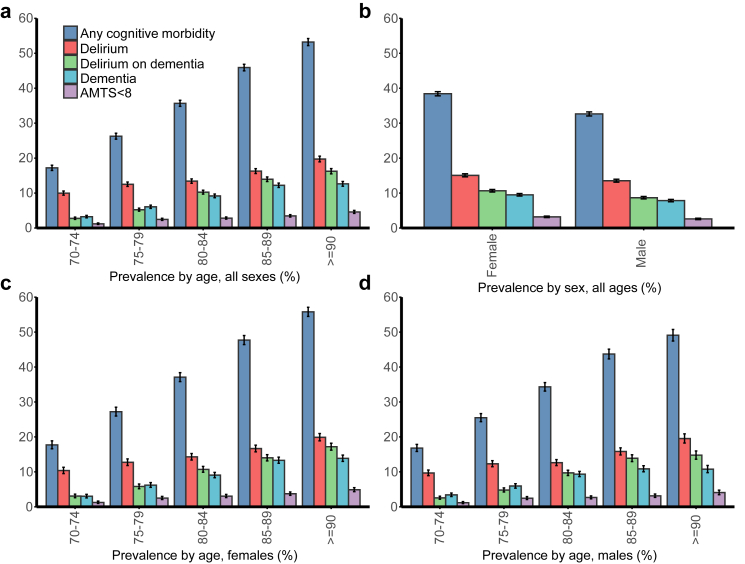
Prevalence of cognitive morbidity hospital-wide stratified separately by age and sex and then stratified by both age and sex together (denominator includes all patients ie both patients with and without a completed cognitive screen).

The prevalence of cognitive morbidity increased linearly with age (*p* trend <0.01) rising threefold from 1665/9675 (17.2%, 16.5–18.0%) of admissions aged 70–74 years to 4864/9140 (53.2%, 52.2–54.2%) of admissions aged ≥90 years (Fig. 1 and Table S5). Delirium in the absence of known dementia was the most prevalent cognitive morbidity subtype in all age groups, ranging from 966/9675 (10.0%, 9.4–10.6%) at age 70–74 years to 1804/9140 (19.7%, 18.9–20.6%) at age ≥90 years. Delirium superimposed on dementia was present in 268/9675 (2.8%, 2.5–3.1%) of admissions aged 70–74 years to 1485/9140 (16.2%, 15.5–17.0%) in those ≥90 years. Dementia in the absence of delirium diagnosis was present in 314/9675 (3.3%; 2.9–3.6%) of admissions aged 70–74 years versus 1155/9140 (12.6%; 12.0–13.3%) of those ≥90 years. Low cognitive test score in the absence of any diagnosed dementia or delirium was the least prevalent across all age groups, ranging from 1.2% (1.0–1.5%; 117/9675) in those 70–74 years to 4.6% (4.2–5.1%; 420/9140) in those ≥90 years. Women had more cognitive morbidity overall than men, especially among those aged ≥80 years (Fig. 1, Table S7).

**Fig. 2:**
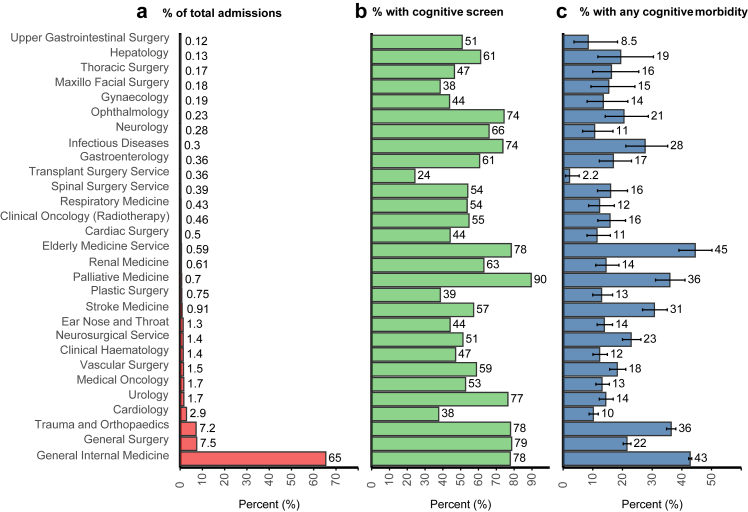
Acute hospital specialty-specific data for percentage of total acute hospital admissions (left panel), percentage with cognitive data (middle panel) and prevalence of cognitive morbidity of any type (denominator includes all patients ie patients with and without a completed cognitive screen).

Screening rates across specialties ranged from 24 to 90% (Fig. 2). Cognitive morbidity was present in patients admitted to all 29 medical and surgical specialties examined (mean age = 74–83 years), with a prevalence exceeding 10% in all except for upper gastrointestinal and transplant surgery (Fig. 2, Table 2). Among medical specialties, the prevalence of cognitive morbidity was highest in geriatric (44.5%, 95% CI 38.8–50.3%; 134/301; mean/SD age = 83/7 years) and general internal medicine (42.8%, 42.3–43.3%; 14,346/33,512; mean/SD age = 83/7), which accounted for 65% (33,512/51,202) of admissions overall and 78.7% (14,346/18,225) of cognitive morbidity hospital-wide. Prevalence was also high in palliative care (36.0%, 31.0–41.2%; 128/356; mean/SD age = 79/6 years), stroke medicine (30.8%, 26.7–35.2%; 144/468; mean/SD age = 82/7 years), infectious disease (27.6%, 20.8–35.6%; n = 42/152; mean/SD age = 82/7 years), hepatology (19.4%, 11.1–31.2%; n = 13/67; mean/SD age = 78/6 years), gastroenterology (16.9%, 12.0–23.3%; n = 31/183; mean/SD age = 78/6 years) and oncology/radiotherapy (15.8%, 11.5–21.3%; 37/234; mean age = 78/5 years).

**Fig. 3:**
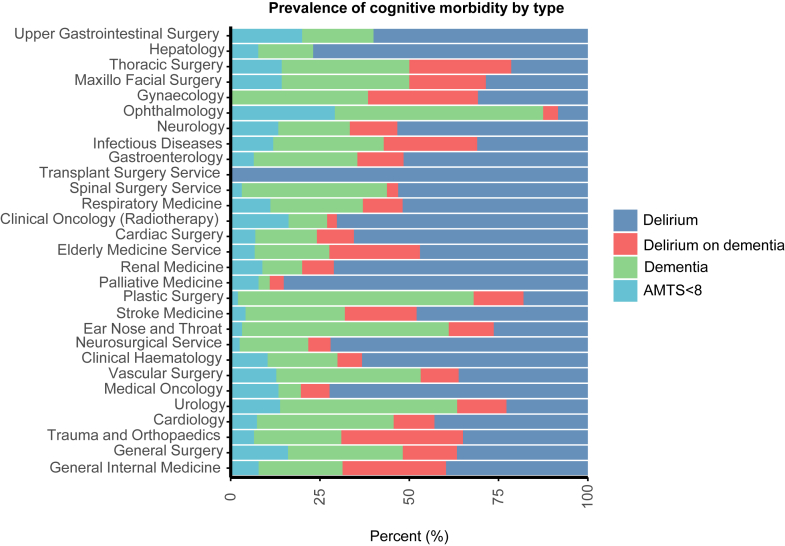
Proportion of cognitive morbidity subtypes (ie delirium, delirium on dementia, dementia and AMTS <8) across specialties. Coloured bars represent the percentage of all cognitive comorbidity for a given specialty.

Among surgical specialties, prevalence of cognitive morbidity was highest in trauma and orthopaedics (36.4%, 34.8–38.0%; 1337/3673; mean/sd age = 83/8 years), neurosurgery (22.9%, 19.9–26.3%; n = 161/702; mean/sd age = 78/6 years) and general surgery (21.5%, 20.2–22.9%; n = 822/3819; mean/sd age = 80/7 years) but was also notable in ophthalmology (20.5%, 13.8–29.2%; 24/117; mean/SD age = 81/7 years), spinal (16.0%, 11.1–22.0%; 32/200; mean/sd age = 78/6 years) and thoracic surgery (16.3%; 9.5–26.1%; 14/86; mean/sd age = 79/7 years). Delirium was the most common cognitive morbidity disorder in 24/29 specialties although delirium superimposed on dementia was rare or absent in hepatology, oncology, upper GI surgery and palliative care where dementia was uncommon (Fig. 3, Table 2). In contrast, dementia alone was the predominant cognitive morbidity subtype in urology, plastics, vascular, ear nose and throat and ophthalmology.

## Discussion

In this large, real-world, hospital-wide cohort, over one third of patients aged 70 years or older with unplanned hospital admission had cognitive morbidity. Delirium, either alone or superimposed on dementia, accounted for nearly three quarters of the cognitive morbidity occurring in one quarter of all admissions. The burden was highest in general (internal) medicine which accounted for over three-quarters of all cognitive morbidity hospital-wide owing to the large number of admissions and high prevalence. Nevertheless, cognitive morbidity was present in all 29 specialties examined although prevalence varied widely from <5% to over 40%.

ORCHARD-EPR provides granular data on cognitive morbidity acquired prospectively as part of mandated cognitive screening in the older acute hospital population and there are no comparable large scale studies to our knowledge. In the cohort overall, 73% of the >50,000 admissions had cognitive data available and there were no exclusion criteria (ie both screened and unscreened patients were included in analyses). In a previous hospital-wide study of delirium enrolling a convenience sample (n = 1507; mean age = 80 years), the prevalence of combined diagnosed and possible delirium was lower at 21% than in our study (24%) but palliative patients and those who could not be assessed for logistical reasons were excluded.^6^ Another study using a large scale electronic hospital data patient cohort of mean age 56 years and algorithmically defined delirium based on nurse administered screening, reported an overall delirium prevalence of 20% although elective as well as emergency patients appeared to have been included.^22^ Dementia prevalence in our study (18%), overlapped with the range reported in previous hospital-wide studies using a variety of ascertainment techniques (7–26%; mean age = 79–84 years, Supplement Literature Review).^11^^,^^12^

Hospital-wide, cognitive morbidity tripled from 17% at age 70–74 years to affect over half of those aged 90 years or older. Delirium was the most common cause at all ages and most patients with delirium (60%) did not have a pre-existing dementia diagnosis. However, dementia, and delirium superimposed on dementia, rose markedly with age and over half of patients with dementia had delirium, consistent with dementia being a strong risk factor.^3^^,^^4^ Dementia was likely under-ascertained however since undiagnosed dementia is common in hospital patients particularly if disease is mild.^10^ Cognitive impairment as defined by the AMT was infrequent (<3%) in the absence of a delirium or dementia diagnosis as seen previously in older medical patients^3^^,^^23^ but some mild cognitive impairment was likely missed owing to the ceiling effect of the AMT.^24^ Women had overall more cognitive morbidity than men, and particularly dementia, consistent with population-based studies.^25^

Cognitive morbidity was ubiquitous throughout the acute hospital specialties we examined, exceeding 10% in all except transplant and upper gastrointestinal surgery. A high prevalence in specialties including geriatric and general (internal) medicine, and trauma and orthopaedics was expected since these specialties have a high proportion of patients with dementia and other risk factors for delirium (eg frailty, multimorbidity) as well as acute precipitants (eg severe illness, infection, trauma). However, the burden of cognitive morbidity was also considerable in specialties less associated with the care of older, frail patients including palliative care, infectious diseases, surgery (general, neurologic, spinal, thoracic), hepatology (where encephalopathy is common), gastroenterology and oncology likely because of a high burden of acute and chronic illness, as well as trauma. Delirium was the most frequent cognitive morbidity diagnosis in almost all specialties usually occurring in the absence of dementia as for the cohort overall likely because high illness acuity is common in emergency admissions and is a strong precipitant of delirium. However, dementia (without delirium) was the most frequent cognitive morbidity subtype in ear, nose and throat, urology, ophthalmology and plastic surgery probably because presentations in these specialties (eg epistaxis, pretibial laceration) are not typically associated with delirium precipitants such as severe illness, infection or metabolic derangement whereas dementia is a common comorbidity.^4^

There are very few previous studies enabling direct comparison of cognitive morbidity prevalence by specialty ascertained through prospective cognitive screening within the same hospital system. In one study using a convenience sample ascertained on a single day, point delirium prevalence was reported for a limited number of specialties. Delirium prevalence was overall lower than in our study but the relative prevalence was similar being highest in geriatric medicine (22%), general medicine (16%) and orthopaedic surgery (15%) and lower in other medicine (10%), stroke (7%) and general surgery (6%).^6^^,^^7^ Regarding dementia, prevalence estimates are only available for broad specialty categories in two studies: internal medicine (21%), trauma (22%), general surgery (13%) and other surgery (12%) in one study^11^ and geriatrics/orthopaedics (42%), medicine (26%) and surgery (12%) in another.^12^

Strengths of our study include the large, inclusive cohort of consecutive, unselected admissions to all four acute hospitals covering a defined region where cognitive screening for patients aged ≥70 years was mandatory irrespective of specialty, and compliance with screening was generally good.^19^^,^^20^ Our study therefore combines the large scale and lack of selection bias of ‘big data’ administrative datasets with the detailed cognitive phenotyping of smaller, prospective studies enabling hospital-wide and specialty-specific estimates of delirium and dementia thereby filling an important evidence gap.

There are some limitations. First, cognitive screening rates were relatively low in some specialties and undetected cognitive morbidity may have resulted in lower measured versus actual prevalence (since we included both screened and unscreened patients in the denominator) although patients with missing cognitive data were generally younger and fitter with fewer risk factors. This may have been compounded by reported low sensitivity of the CAM^26^ although we have previously shown that our holistic delirium screening approach results in a measured delirium prevalence approaching the true delirium prevalence.^19^^,^^20^ Second, we defined delirium using both certain and uncertain diagnoses building on our previous work and that of others^6^^,^^19^^,^^20^ but restriction to certain cases did not impact key findings including that delirium was more prevalent than dementia. Third, it was not possible to distinguish between delirium present on admission and incident delirium (arising during admission) despite implications for clinical practice, although previous studies have showed that most delirium in acute settings is present on admission.^3^ Fourth, our study was restricted to patients aged ≥70 years where cognitive screening was mandatory, but cognitive morbidity also occurs in younger hospital patients: delirium affects around 10% of those aged 50–64 and ∼15% of those 65–69 years in acute general medicine.^3^ Fifth, our findings may not be generalisable to other settings with differing case-mix and care pathways.^27^ In particular, the proportion of in-patients with cognitive morbidity may be lower in countries where the number of hospital beds per capita is higher and admission avoidance services are less developed than in the UK.

Our study has several implications for service planning and policy where to date, delirium has received comparatively less attention than dementia.^4^ Cognitive morbidity affected one third of the older acute hospital population and delirium without a comorbid dementia diagnosis was most common, whereas dementia alone was relatively infrequent. Our findings support current guidance for routine delirium screening which should be delivered hospital-wide across all acute specialties.^1^ The relative prevalence of overall cognitive morbidity and of the different cognitive morbidity subtypes by specialty lends face validity to ORCHARD-EPR data and suggests that screening was performed reasonably accurately. Nevertheless, variations in screening rates across specialties occurred and may have been the result of differences in service pressures, the perceived value of screening and clinician engagement but compliance was higher in services with perioperative physician support (eg general surgery-79% screened) or incentivised screening (trauma and orthopaedics-78% screened). Although a handful of specialties had low prevalence of delirium with dementia being more common, it would nevertheless seem appropriate to maintain a consistent screening approach across all acute settings given the importance of detecting delirium and avoiding impact on compliance.

Management of delirium is often challenging owing to the fluctuating course, unpredictable cognitive prognosis, issues around capacity and consent,^28^ and risk of poor outcomes including delayed discharge.^3^^,^^4^ Our study shows the need for skilled multidisciplinary team care across multiple acute services that could be provided by a combination of ward-based teams including perioperative physicians where appropriate and mobile “out-reach” or liaison teams in low prevalence specialties. Training non-geriatrician clinicians to provide comprehensive geriatric assessment may be less effective.^29^

Our findings also have implications for dementia diagnosis and prevention. Delirium is a known risk factor for dementia^4^^,^^30^, ^31^, ^32^ and since hospitalisation is common in older people, older hospital patients constitute a group of importance to dementia prevention at the population level. Notably, those with previously normal cognition have the most to lose from delirium as they experience greater relative impact underscoring the importance of targeting interventions and research to develop treatments to prevent cognitive decline in this group.^3^^,^^30^, ^31^, ^32^ Further, around half of older hospital patients with dementia are undiagnosed and our cognitive screen combining delirium screening and a cognitive test will identify those in whom in-hospital assessment for possible underlying dementia should be considered.^33^^,^^34^

In conclusion, over one-third of all patients aged 70 years or older with unplanned hospital admission had cognitive morbidity with delirium being the most frequent subtype, usually occurring in the absence of pre-existing dementia diagnosis. Cognitive morbidity was more likely with increasing age and female sex and occurred in all acute specialties although prevalence varied. Our findings should inform healthcare provision and policy including the need for routine delirium screening across all acute specialties and multidisciplinary support allocated according to the likely cognitive morbidity burden.

## Contributors

ELB cleaned and assembled the ORCHARD-EPR data, performed analyses and drafted the manuscript including creating the figures. SCS co-led OUHFT cross divisional cognitive screening compliance and provided critical input to the manuscript. SSi co-led EHR implementation of the cognitive screen and supported screening compliance. SSh provided supervision and critical input into the analyses and manuscript. STP provided supervision, obtained funding, led the development, implementation and performance feedback of the cognitive screen, obtained raw data from the OUHFT Information team, and was responsible for study conception, study design and drafting of the manuscript. ELB and STP had full access and verified the underlying data used in this study. All authors accept responsibility to submit for publication.

## Data sharing statement

At present, ethics approval restrictions limit the use of data to researchers within the University of Oxford or OUHFT. Requests for data should be made to STP and will be considered on a case by case basis.

## Declaration of interests

ELB has received funding from the Rhodes Trust and the Canadian Institutes of Health Research (CIHR) and for travel from the Guarantors of Brain and Alzheimer's Research UK. The other authors have no conflicts of interest to declare.

## References

[1] Hopper A. (2021).

[2] Boucher E.L., Gan J.M., Rothwell P.M., Shepperd S., Pendlebury S.T. (2023). Prevalence and outcomes of frailty in unplanned hospital admissions: a systematic review and meta-analysis of hospital-wide and general (internal) medicine cohorts. eClinicalMedicine.

[3] Gan J., Boucher E.L., Lovett N.G., Roche S., Smith S.C., Pendlebury S.T. (2025). Observational, longitudinal study of delirium in over 1800 consecutive unselected acute general (internal) medicine patients: age-specific occurrence, associated factors, and outcomes to 10-years. Lancet Healthy Longev.

[4] Wilson J.E., Mart M.F., Cunningham C. (2020). Delirium. Nat Rev Dis Primers.

[5] Harwood R., BGS Dementia and Related Disorders SIG, British Geriatrics Society (2022).

[6] Geriatric Medicine Research Collaborative (2019). Delirium is prevalent in older hospital inpatients and associated with adverse outcomes: results of a prospective multi-centre study on World Delirium Awareness Day. BMC Med.

[7] Geriatric Medicine Research Collaborative (2021). Retrospective delirium ascertainment from case notes: a retrospective cohort study. BMJ Open.

[8] Ibitoye T., So S., Shenkin S.D. (2023). Delirium is under-reported in discharge summaries and in hospital administrative systems: a systematic review. Delirium.

[9] Pendlebury S.T., Lovett N.G., Thomson R.J., Smith S.C. (2020). Impact of a system-wide multicomponent intervention on administrative diagnostic coding for delirium and other cognitive frailty syndromes: observational prospective study. Clin Med.

[10] Sampson E.L., Blanchard M.R., Jones L., Tookman A., King M. (2009). Dementia in the acute hospital: prospective cohort study of prevalence and mortality. Br J Psychiatry.

[11] Bickel H., Hendlmeier I., Heßler J.B. (2018). The prevalence of dementia and cognitive impairment in hospitals. Dtsch Arztebl Int.

[12] Timmons S., Manning E., Barrett A. (2015). Dementia in older people admitted to hospital: a regional multi-hospital observational study of prevalence, associations and case recognition. Age Ageing.

[13] Boucher E., Jell A., Singh S. (2024). Protocol for the development and analysis of the Oxford and Reading Cognitive Comorbidity, Frailty and Ageing Research Database-electronic Patient Records (ORCHARD-EPR). BMJ Open.

[14] Inouye S.K., van Dyck C.H., Alessi C.A., Balkin S., Siegal A.P., Horwitz R.I. (1990). Clarifying confusion: the confusion assessment method. A new method for detection of delirium. Ann Intern Med.

[15] Hodkinson H.M. (1972). Evaluation of a mental test score for assessment of mental impairment in the elderly. Age Ageing.

[16] The Royal College of Physicians (2012).

[17] NHS Digital (2020).

[18] Gilbert T., Neuburger J., Kraindler J. (2018). Development and validation of a hospital frailty risk score focusing on older people in acute care settings using electronic hospital records: an observational study. Lancet.

[19] Boucher E.L., Gan J.M., Lovett N.G., Smith S.C., Shepperd S., Pendlebury S.T. (2025). Implementation of delirium screening at scale in older patients with emergency hospital admission. JAMA Intern Med.

[20] Boucher E.L., Gan J.M., Lovett N.G., Smith S.C., Shepperd S., Pendlebury S.T. (2025). Delirium prevalence, diagnostic uncertainty and outcomes in ORCHARD-EPR: validation against prospective reference cohorts. Age Ageing.

[21] Bellelli G., Morandi A., Davis D.H. (2014). Validation of the 4AT, a new instrument for rapid delirium screening: a study in 234 hospitalised older people. Age Ageing.

[22] Zipser C.M., Spiller T.R., Hildenbrand F.F. (2022). Discharge destinations of delirious patients: findings from a prospective cohort study of 27,026 patients from a large health care system. J Am Med Dir Assoc.

[23] Reynish E.L., Hapca S.M., De Souza N., Cvoro V., Donnan P.T., Guthrie B. (2017). Epidemiology and outcomes of people with dementia, delirium, and unspecified cognitive impairment in the general hospital: prospective cohort study of 10,014 admissions. BMC Med.

[24] Emery A., Wells J., Klaus S.P., Mather M., Pessoa A., Pendlebury S.T. (2020). Underestimation of cognitive impairment in older inpatients by the abbreviated mental test score versus the montreal cognitive assessment: cross-sectional observational study. Dement Geriatr Cogn Dis Extra.

[25] Matthews F.E., Arthur A., Barnes L.E. (2013). Medical Research Council Cognitive Function and Ageing Collaboration. A two-decade comparison of prevalence of dementia in individuals aged 65 years and older from three geographical areas of England: results of the Cognitive Function and Ageing Study I and II. Lancet.

[26] Penfold R.S., Squires C., Angus A. (2024). Delirium detection tools show varying completion rates and positive score rates when used at scale in routine practice in general hospital settings: a systematic review. J Am Geriatr Soc.

[27] Elias T.C.N., Bowen J., Hassanzadeh R., Lasserson D.S., Pendlebury S.T. (2021). Factors associated with admission to bed-based care: observational prospective cohort study in a multidisciplinary same day emergency care unit (SDEC). BMC Geriatr.

[28] Gan J.M., Riley J., Basting R., Demeyere N., Pendlebury S.T. (2023). Decision-making capacity in older medical in-patients: frequency of assessment and rates of incapacity by decision-type and underlying brain/mind impairment. Age Ageing.

[29] Ellis G., Gardner M., Tsiachristas A. (2017). Comprehensive geriatric assessment for older adults admitted to hospital. Cochrane Database Syst Rev.

[30] Pendlebury S.T., Luengo-Fernandez R., Seeley A. (2024). Infection, delirium, and risk of dementia in patients with and without white matter disease on previous brain imaging: a population-based study. Lancet Healthy Longev.

[31] Tsui A., Searle S.D., Bowden H. (2022). The effect of baseline cognition and delirium on long-term cognitive impairment and mortality: a prospective population-based study. Lancet Healthy Longev.

[32] Krogseth M., Davis D., Jackson T.A. (2023). Delirium, neurofilament light chain, and progressive cognitive impairment: analysis of a prospective Norwegian population-based cohort. Lancet Healthy Longev.

[33] Penfold R.S., Bowman E., Vardy E.R.L.C. (2025). Using scores from the 4AT delirium detection tool as an indicator of possible dementia: a study of 75 221 older adult hospital admissions. Age Ageing.

[34] Pendlebury S.T., Wadling S., Silver L.E., Mehta Z., Rothwell P.M. (2011). Transient cognitive impairment in TIA and minor stroke. Stroke.

