# Supplementary material for: Prevalence of cognitive morbidity including delirium in 51,202 emergency hospital admissions across 29 medical and surgical specialties in ORCHARD-EPR: a cross-sectional study
Source: eClinicalMedicine. 2025 Nov 24;90:103641. doi: 10.1016/j.eclinm.2025.103641 (PMC12686935; doi:10.1016/j.eclinm.2025.103641)
Supplement: Supplementary Material [file mmc1.docx]

**Supplementary Material**

**Prevalence of cognitive morbidity including delirium in 51,202 emergency hospital admissions across 29 medical and surgical specialties in ORCHARD-EPR: a cross-sectional study**

Boucher EL, BHSc^1^; Smith SC, Singh S, Shepperd S, DPhil^3^; Pendlebury ST, FRCP, DPhil^1,2,4^

^1^Wolfson Centre for Prevention of Stroke and Dementia, Wolfson Building, Nuffield Department of Clinical Neurosciences, University of Oxford, UK

^2^Departments of Acute General (Internal) Medicine and Geratology, Oxford University Hospitals NHS Foundation Trust, UK

^3^Nuffield Department of Population Health, University of Oxford, UK

^4^NIHR Biomedical Research Centre, Oxford University Hospitals NHS Foundation Trust, UK

Address correspondence to: Professor Sarah Pendlebury, Wolfson Centre for Prevention of Stroke and Dementia, Wolfson Building, John Radcliffe Hospital, Oxford OX3 9DU

Email: [sarah.pendlebury@ndcn.ox.ac.uk](mailto:sarah.pendlebury@ndcn.ox.ac.uk)

Telephone: +44 1865 231603

Table of contents Page

Box S1. Uses of cognitive morbidity estimates, hospital-wide and by specialty 2

Systematic Literature Review 3

Table ‎S1. Studies reporting delirium prevalence in acute hospital-wide cohorts 4

Table S2. Studies reporting dementia prevalence in acute hospital-wide cohorts 5

Supplementary Methods 7

Study setting and cognitive screening 7

ICD-10 codes used to identify delirium and dementia 11

Supplementary Results 12

Table S3. Characteristics of ORCHARD-EPR specialty groups 12

Table S4. Characteristics of people with cognitive data vs missing cognitive data 13

Table S5. Cognitive morbidity prevalence by age group (delirium defined as certain and uncertain delirium combined) 14

Table S6. Cognitive morbidity prevalence by age group including certain delirium only 15

Table S7. Prevalence of cognitive morbidity subtypes by age and sex for the acute hospital-wide cohort. 16

Figure S1. Flow chart showing the derivation of the study cohort from ORCHARD-EPR 17

Figure S2. Pathways to cognitive morbidity diagnosis and ascertainment inn ORCHARD-EPR 18

**Box S1. Uses of cognitive morbidity estimates, hospital-wide and by specialty**

**Planning clinical services**

• Evaluate consent processes;

• Target cognitive screening programmes to relevant services;

• Inform the implementation of mobile versus ward-based frailty teams (i.e., ward-based teams in specialties with a high prevalence of cognitive morbidity and mobile teams in specialties with lower prevalence);

• Inform staffing levels and skill-mix.

**Health education and training**

• Develop cognitive morbidity health education curricular objectives;17

• Inform the development of skills training standards (e.g., Health Education England’s Dementia Training Standards Framework).18

**Research**

• Inform the choice of funding priorities by identifying the burden of cognitive morbidity in a hospital setting;

• Inform research ethics review and consent processes;

• Inform research design including target population, selection criteria and generalisability.

**Systematic Literature Review**

We reviewed observational hospital-wide studies reporting delirium and dementia prevalence in adults >18 years with unplanned admission.

Search: MEDLINE and EMBASE were searched from inception to 28/06/2025 using MeSH and Emtree terms and text keywords related to [Delirium], [Dementia], [Cognitive Impairment], [Cognitive Defect/Decline], [Cognitive Spectrum Disorder] and [Hospitalisation]. We also searched reference lists of relevant reviews.

Eligibility: Studies were included if they (i) reported delirium or dementia ascertained using validated screening tools or diagnosed clinically in hospital (within the first three days of admission, unless assessed retrospectively); (ii) included mostly unplanned admissions (>70% as stated by the study author or inferred from the study description); and (iii) reported data for hospital-wide admissions. Studies were excluded if they were (i) conducted in outpatient, emergency department, short-stay, geriatric, rehabilitation, mixed or specialty-specific settings; (ii) conducted in non-representative populations (diagnosis, clinical trial, risk profile, excluded patients with dependency, etc.); (iii) n<100; (iv) did not report cohort age and/or (v) the full text was not available in English.

Screening and data extraction: We performed title-abstract (n=2,223) and full-text screening. We extracted data including study and participant characteristics, recruitment method, delirium/dementia ascertainment method and prevalence, including by specialty where available.

Results: Included studies are shown in Tables S1 and S2. There were only two studies reporting delirium occurrence hospital-wide and two studies reporting dementia prevalence although sample sizes were all greater than 500. In all four studies, patients had a mean age of around 80 years. Methodology varied across studies with both prospective and retrospective approaches employed and convenience sampling versus consecutive ascertainment. Some researchers ascertained delirium or dementia on one day only whereas others captured data from the entire admission episode for each patient. Notably. some studies used retrospective chart review to ascertain delirium or ICD-10 diagnostic coding to ascertain dementia both of which are known to have lower sensitivity than prospective diagnostic methods.

Table ‎S1. Studies reporting delirium prevalence in acute hospital-wide cohorts

| **Study** | **Country** | **N** | **Sampling  (% enrolled)** | **Mean age, years** | **Female (%)** | **Exclusion criteria** | **Delirium ascertainment** | **Details of ascertainment** | **Delirium prevalence (%)** | **Breakdown by specialty** |
| --- | --- | --- | --- | --- | --- | --- | --- | --- | --- | --- |
| GMRC, (2019)^1^ | UK | 1,507 | Convenience (63%) | 80 | 54% | Age <65 years, ICU, palliative, logistics | DSM-V if 4AT ≥4/12 | Assessed on day of study by research team (P) | Point prevalence=15% (21% including possible delirium) | Acute medicine, geriatric medicine, other medical, stroke, general/other surgery, orthopaedic |
| GMRC (2021)^2^ | UK | 626 | Retrospective (NA) | 80 | 54% | Age <65 years, LoS <2 days, elective | Chart review | Done by research team (U) | 21% | Acute medicine, geriatric medicine, other medical, stroke, general/other surgery, orthopaedic |

**Legend:**. DSM = Diagnostic and Statistical Manual of Mental Disorders. GMRC=Geriatric Medicine Research Collaborative. ICU = Intensive care unit. LoS=Length of stay. NA = not available. P = Prospective ascertainment. U = Unclear methodology. Ascertainment tools: **Note:** Studies with n<100 were excluded.

Table S2. Studies reporting dementia prevalence in acute hospital-wide cohorts

| **Study (n=10)** | **Country** | **N** | **Sampling  (% enrolled)** | **Age, years** | **Female (%)** | **Exclusion criteria** | **Dementia ascertainment** | **Details of ascertainment** | **Prevalence (%)** | **Breakdown by specialty** |
| --- | --- | --- | --- | --- | --- | --- | --- | --- | --- | --- |
| Bickel (2018)^3^ | Germany | 1,439 | Entire wards on single day (58%) | 79 | 54% | Age <65 years, rehabilitation, geriatrics, neurology, psychiatry, ICU or isolation ward; moribund; non-German speaking; no patient/proxy consent | DSM-IV | Assessed by research team (inferred) (P) | 18% (DSM-IV); 6.8% (medical records) | Internal medicine, general surgery, trauma, other surgery |
| Timmons (2015)^4^ | Ireland | 598 | Consecutive (88%) | 80 | 51% | <70 years, day-cases,  moribund,  refused | DSM-IV or known dementia | If MMSE<27/30 or unavailable and no previous diagnosis, then diagnosed by study author or expert panel (P) | 25% | Medicine, surgery, geriatrics/orthopaedics |

**Legend:** NA = not available. (P) = Prospective ascertainment. (C) = Routine clinical data. (A) = Routine administrative data. Ascertainment tools: DSM = Diagnostic and Statistical Manual of Mental Disorders. ICD-10 = International Classification of Diseases, Tenth Revision.

**Note:** Studies with n<100 were excluded.

**References**

1. Geriatric Medicine Research Collaborative. Delirium is prevalent in older hospital inpatients and associated with adverse outcomes: results of a prospective multi-centre study on World Delirium Awareness Day. BMC Med. 2019 Dec 14;17(1):229.

2. Geriatric Medicine Research Collaborative. Retrospective delirium ascertainment from case notes: a retrospective cohort study. BMJ Open. 2021 May 28;11(5):e042440.

3. Bickel H, Hendlmeier I, Heßler JB, et al. The Prevalence of Dementia and Cognitive Impairment in Hospitals. *Deutsches Aerzteblatt Online* 2018; 115(44): 733-40.

4. Timmons S, Manning E, Barrett A, et al. Dementia in older people admitted to hospital: a regional multi-hospital observational study of prevalence, associations and case recognition. *Age Ageing* 2015; 44(6): 993-9.

**Supplementary Methods**

**Study setting and cognitive screening**

The Oxford University Hospitals NHS Foundation Trust provides all acute secondary care services to the entire population of the Oxfordshire region (~800,000) and is the sole provider of acute services. The Oxfordshire population is similar to the background population of England as a whole (~95% Caucasian at age >65 years) with an urban and rural mix although slightly older and less deprived but all levels of deprivation are represented. In 2015, Oxford University Hospitals went paperless with the roll-out of electronic health records (EHRs), termed in most NHS hospitals as electronic patient records (EPRs) thereafter used to record all healthcare encounters including clinical information, observations performed by nursing staff, laboratory investigations and non-laboratory diagnostic tests.

In designing our cognitive screen, we chose the Confusion Assessment Method (CAM, short version) as the most validated pragmatic delirium screen available at the time.^1^ However, the CAM is a screen rather than a diagnostic test and has been reported to have suboptimal sensitivity in some studies,^2^ and the clerking resident doctor is therefore required to document delirium diagnosis as part of a holistic assessment made on the basis of all available information following the history and examination and informant interview where necessary. We combined the CAM with the 10-point abbreviated mental test (AMT) to identify and quantify the severity of cognitive deficits including in those without delirium having demonstrated that the AMT was more feasible to do routinely than the mini-mental-state-examination (MMSE) in all older patients admitted to an acute medicine service.^3^ We validated the AMT against the Montreal Cognitive Assessment (MoCA) in two studies which showed it was specific for moderate/severe impairment although insensitive to milder impairments.^4,5^ Notably, the screen was designed to be completed whether or not the AMT was feasible to perform ie it was completed on testable and untestable patients. Reasons for untestability were recorded via a drop down list in the EPR cognitive screening proforma.

The finalised cognitive screen therefore included the AMT and two questions to document delirium or dementia diagnosis:

- The 10-point AMT, or reason for untestability recorded in a drop down multichoice list (eg too unwell, aphasia) including “other” where the problem was not listed with the option to record this using free text entry;
- “Does the patient have a known diagnosis of dementia?”
- “Does the patient have delirium?” informed by the CAM (individual CAM items were not recorded).

The assessor was required to record their answer to the delirium question with one of the following: “yes”, “no” or “uncertain”. We enabled clinicians to record diagnostic uncertainty in the diagnosis of delirium to reflect clinical realities including difficulty in establishing recent behavioural change (a key component of the DSM criteria for delirium and therefore the CAM). The proforma was designed for completion even in untestable patients and partial completion was not possible.

The cognitive screen was delivered initially via a paper clerking proforma (2012-2015) and from 2015, via a bespoke structured powerform (Cerner Millenium) integrated into the EPR.^6,7^  A screenshot of the proforma is shown in Supplementary Figure The EPR cognitive screen is triggered automatically on-admission for all patients aged >70 years with unplanned admission to ensure completion and also because most (80%) delirium in unplanned hospital admissions is present on admission rather than being incident during admission.^3^ Roll-out across all four general hospitals covering the Oxfordshire region (population ~800,000) was supported by a multicomponent intervention as described previously including staff training and performance feedback.^8^ Implementation and compliance with screening was further driven by national requirements including dementia screening targets (2013-2020) and thereafter the GIRFT guidance.^9,10^

Individual EPR data including the individual item datafields in the EPR cognitive screen were extracted by the hospital information analysts and entered by the research team into the Oxford Cognitive Comorbidity, Frailty and Ageing Research Database-Electronic Patient Records (ORCHARD-EPR) for analysis.^8^ Diagnostic (ICD-10) codes applied by the hospital administrative coding team after patient discharge were also extracted and entered into the database. ICD-10 diagnostic codes were included to supplement the cognitive screening results where free text recording of delirium had enabled the coding team to apply a code for delirium in the absence of a completed screen or where the screen had been completed but was initially negative for delirium or delirium was recorded as uncertain. Sensitivity for ICD-10 coding for delirium in our institution is good and considerably higher than in most other routinely acquired administrative datasets and specificity approaches 100%.^6^

Subsequent evaluation following EPR implementation of the cognitive screen has shown that the screening process achieves accurate identification and documentation of delirium at scale with the measured prevalence (of combined certain and uncertain delirium diagnosis) approaching the true prevalence.^11,12^ Certain and uncertain delirium have been shown to have very similar characteristics and outcomes hence were combined for the current study.^12^

Charlson co-morbidity index

The Charlson Index (CCI) is a composite measure of comorbidity which contains 17 differently weighted categories of conditions such as cancer, heart failure, lung disease etc. A score of 0 indicates that none of the 17 conditions is present. The higher the score, the greater the comorbidity burden and the lower the 10-year survival. There are numerous updated weightings and other adaptations of the score. We used the version currently in use in the UK National Health service (NHS).^13,14^

Hospital Frailty Risk Score

The Hospital Frailty Risk Score (HFRS) was developed to identify frailty patients at risk of poor outcomes in large administrative datasets. The HFRS is calculated from a large number of frailty related ICD-10 diagnostic codes and is therefore subject to variation according to the accuracy of diagnostic coding in a given institution. However, despite this variation, the HFRS identifies vulnerable groups at risk and correlates with other measures of frailty and exhibits a dose response effect.^15,16^ For the current study, the HFRS was calculated without cognitive diagnoses since prevalence of cognitive morbidity was the focus of the analysis.

**References**

1. Inouye SK, van Dyck CH, Alessi CA, Balkin S, Siegal AP, Horwitz RI. Clarifying confusion: the confusion assessment method. A new method for detection of delirium. Ann Intern Med.1990;113:941-8.

2. Penfold RS, Squires C, Angus A, Shenkin SD, Ibitoye T, Tieges Z, Neufeld KJ, Avelino-Silva TJ, Davis D, Anand A, Duckworth AD, Guthrie B, MacLullich AMJ. Delirium detection tools show varying completion rates and positive score rates when used at scale in routine practice in general hospital settings: A systematic review. J Am Geriatr Soc. 2024;72:1508-1524.

3. Pendlebury ST, Lovett NG, Smith SC, Dutta N, Bendon C, Lloyd-Lavery A, Mehta Z, Rothwell PM. Observational, longitudinal study of delirium in consecutive unselected acute medical admissions: age-specific rates and associated factors, mortality and re-admission. BMJ Open 2015;5(11):e007808.

4. Emery A, Wells J, Klaus SP, Mather M, Pessoa A, Pendlebury ST: Underestimation of Cognitive Impairment in Older Inpatients by the Abbreviated Mental Test Score versus the Montreal Cognitive Assessment: Cross-Sectional Observational Study. Dement Geriatr Cogn Disord Extra 2020;**10**:205-215.

5. Pendlebury ST, Klaus SP, Mather M, de Brito M, Wharton RM. Routine cognitive screening in older patients admitted to acute medicine: abbreviated mental test score (AMTS) and subjective memory complaint versus Montreal Cognitive Assessment and IQCODE. Age Ageing 2015;44:1000-5

6. Boucher EL, Gan JM, Lovett NG, Smith SC, Shepperd S, Pendlebury ST. Implementation of Delirium Screening at Scale in Older Patients With Emergency Hospital Admission. JAMA Intern Med. 2025 May 27:e251128.

7. Boucher E, Jell A, Singh S, et al. Protocol for the Development and Analysis of the Oxford and Reading Cognitive Comorbidity, Frailty and Ageing Research Database-Electronic Patient Records (ORCHARD-EPR). BMJ Open 2024;14:e085126.

8. Pendlebury ST, Lovett NG, Thomson RJ, Smith SC. Impact of a system-wide multicomponent intervention on administrative diagnostic coding for delirium and other cognitive frailty syndromes: observational prospective study. Clin Med (Lond) 2020;20:454-464.

9. NHS England. Statistics: Dementia Assessment and Referral 2018-2019. No date. <https://www.england.nhs.uk/statistics/statistical-work-areas/dementia/dementia-assessment-and-referral-2018-19>.

10. Hopper A. Geriatric Medicine: GIRFT Programme National Specialty Reports. NHS England: Getting It Right First Time 2021.

11. Boucher EL, et al. Implementation of Delirium Screening at Scale in Older Patients With Emergency Hospital Admission. JAMA Intern Med. 2025; 27:e251128.

12. Boucher EL, Gan J, Lovett NG, Smith SC, Shepperd S, Pendlebury ST. Delirium prevalence, diagnostic uncertainty and outcomes in ORCHARD-EPR: validation against prospective reference cohorts. Age Ageing, in press

13. Charlson ME, Pompei P, Ales KL, MacKenzie CR. "A new method of classifying prognostic comorbidity in longitudinal studies: development and validation". Journal of Chronic Diseases 1987; 40:373–383

14. NHS Digital. IAP00385 Summary Hospital-level Mortality Indicator (SHMI). Leeds: NHS Digital 2020; 81-84.

15. Gilbert T, Neuburger J, Kraindler J, et al. Development and validation of a Hospital Frailty Risk Score focusing on older people in acute care settings using electronic hospital records: an observational study. Lancet. 2018;391:1775-82.

16. Boucher EL, Gan JM, Rothwell PM, Shepperd S, Pendlebury ST. Prevalence and outcomes of frailty in unplanned hospital admissions: a systematic review and meta-analysis of hospital-wide and general (internal) medicine cohorts. EClinicalMedicine. 2023;59:101947.

**ICD-10 codes used to identify delirium and dementia**

ICD-10 codes for delirium and dementia were used as described previously and included the following:

- F00X - Dementia in Alzheimer Disease
- F01X - Vascular dementia
- F02X - Dementia in other diseases classified elsewhere
- F03X - Unspecified dementia
- G30X - Alzheimer disease
- F051 - Delirium superimposed on dementia
- F107 - Mental and behavioural disorders due to use of alcohol, residual and late-onset psychotic disorder
- F050  - Delirium not superimposed on dementia, so described
- F051 – Delirium superimposed on dementia
- F058 – Other delirium
- F059 - Delirium, unspecified

Supplementary Tables

Table S3. Characteristics of selected specialty groups in ORCHARD-EPR

| Variable | Overall,^1^  N = 51,202 | General Medicine,^1^  N = 33,512 | General Surgery,^1^  N = 3,819 | Specialty Medicine,^1,4^  N =5,613 | Specialty  Surgery,^1,5^  N =4,585 | Trauma and Orthopedics,^1^  N =3,673 | p-value^2^ |
| --- | --- | --- | --- | --- | --- | --- | --- |
| Age | 82 (7) | 83 (7) | 80 (7) | 79 (6) | 79 (7) | 83 (8) | <0.0001 |
| Female sex^3^ | 51% | 52% | 52% | 42% | 38% | 67% | <0.0001 |
| Care home resident | 15% | 18% | 7.6% | 7.8% | 6.3% | 16% | <0.0001 |
| CCI | 11 (10) | 12 (10) | 7 (9) | 11 (9) | 8 (9) | 9 (11) | <0.0001 |
| HFRS | 8 (6) | 9 (6) | 4 (4) | 5 (5) | 5 (5) | 11 (8) | <0.0001 |
| Abnormal NEWS^3^ | 27% | 34% | 13% | 21% | 9.5% | 13% | <0.0001 |
| Data on cognition | 73% | 78% | 79% | 54% | 54% | 78% | <0.0001 |
| ^1^Mean (SD); n / N (%) | | | | | | | |
| ^2^Kruskal-Wallis rank sum test (continuous data); Pearson's Chi-squared test (nominal data) | | | | | | | |
| ^3^Sex missing for n=508; NEWS missing for n=961 | | | | | | | |
| ^4^ The largest specialty medicine services were cardiology (n=1,462), medical oncology (n=853) and clinical haematology (n=708), which comprised over half of admissions within the group. | | | | | | | |
| ^5^ The largest specialty surgical services were urology (n=856), vascular surgery (n=773) and the neurosurgical service (n=702), which comprised over half of admissions within the group. | | | | | | | |

Table S4. Characteristics of people with vs without cognitive data in ORCHARD-EPR

| **Characteristic** | **No cognitive data,**  **N = 13,724^1^** | **Yes cognitive screen and/or ICD codes,**  **N = 37,478^1^** | **p-value^2^** |
| --- | --- | --- | --- |
| Age, mean (SD) | 80 (7) | 83 (7) | <0.0001 |
| Female sex* | 6,511 (48) | 19,347 (52) | <0.0001 |
| Care home resident | 1,545 (11) | 9,396 (25) | <0.0001 |
| CCI | 8 (8) | 12 (10) | <0.0001 |
| HFRS | 5 (4) | 9 (7) | <0.0001 |
| Abnormal NEWS score* | 3,621 (26) | 10,951 (29) | <0.0001 |

| ^1^Mean (SD); n / N (%) |
| --- |
| ^2^Wilcoxon rank sum test; Pearson's Chi-squared test  *Missing data: Sex missing for 147 with no cognitive data and 361 with cognitive data. NEWS score missing for 206 with no cognitive data and 283 with cognitive data |

Table S5. Cognitive morbidity prevalence by age group (delirium defined as certain and uncertain delirium combined)

| **Age group** | **Delirium only** | **Delirium on dementia** | **Dementia only** | **AMTS<8, no known dementia or delirium** | **Any cognitive frailty** |
| --- | --- | --- | --- | --- | --- |
| **70-74** | 966/9,675  10.0% [9.4-10.6%] | 268/9,675  2.77% [2.46-3.12%] | 314/9,675  3.25% [2.91-3.62%] | 117/9,675  1.21% [1.01-1.45%] | 1,665/9,675  17.2% [16.5-18.0%] |
| **75-79** | 1,279/10,216  12.5% [11.9-13.2%] | 534/10,216  5.23% [4.81-5.68%] | 619/10,216  6.06% [5.61-6.54%] | 252/10,216  2.47% [2.18-2.79%] | 2,684/10,216  26.3% [25.4-27.1%] |
| **80-84** | 1,533/11,424  13.4% [12.8-14.1%] | 1,171/11,424  10.3% [9.70-10.8%] | 1,049/11,424  9.18% [8.66-9.73%] | 324/11,424  2.84% [2.54-3.16%] | 4,077/11,424  35.7% [34.8-36.6%] |
| **85-89** | 1,750/10,747  16.3% [15.6-17.0%] | 1,499/10,747  13.9% [13.3-14.6%] | 1,313/10,747  12.2% [11.6-12.9%] | 373/10,747  3.47% [3.14-3.84%] | 4,935/10,747  45.9% [45.0-46.9%] |
| **>=90** | 1,804/9,140  19.7% [18.9-20.6%] | 1,485/9,140  16.2% [15.5-17.0%] | 1,155/9,140  12.6% [12.0-13.3%] | 420/9,140  4.60% [4.18-5.05%] | 4,864/9,140  53.2% [52.2-54.2%] |
| **Overall** | 7,332/51,202  14.3% [14.0-14.6%] | 4,957/51,202  9.68% [9.43-9.94%] | 4,450/51,202  8.69% [8.45-8.94%] | 1,486/51,202  2.90% [2.76-3.05%] | 18,225/51,202  35.6% [35.2-36.0%] |

Table S6. Cognitive morbidity prevalence by age group including certain delirium only

| **Age group** | **Delirium only** | **Delirium on dementia** | **Dementia only** | **AMTS<8, no known dementia or delirium** | **Any cognitive frailty** |
| --- | --- | --- | --- | --- | --- |
| **70-74** | 519/9,675  5.36% [4.93-5.84%] | 151/9,675  1.56% [1.33-1.83%] | 431/9,675  4.45% [4.06-4.89%] | 233/9,675  2.41% [2.12-2.74%] | 1,334/9,675  13.8% [13.1-14.5%] |
| **75-79** | 718/10,216  7.03% [6.54-7.55%] | 318/10,216  3.11% [2.79-3.47%] | 835/10,216  8.17% [7.65-8.73%] | 435/10,216  4.26% [3.88-4.67%] | 2,306/10,216  22.6% [21.8-23.4%] |
| **80-84** | 924/11,424  8.09% [7.60-8.61%] | 693/11,424  6.07% [5.64-6.52%] | 1,527/11,424  13.4% [12.8-14.0%] | 525/11,424  4.60% [4.22-5.00%] | 3,669/11,424  32.1% [31.3-33.0%] |
| **85-89** | 1,044/10,747  9.71% [9.16-10.3%] | 909/10,747  8.46% [7.94-9.00%] | 1,903/10,747  17.7% [17.0-18.4%] | 639/10,747  5.95% [5.51-6.41%] | 4,495/10,747  41.8% [40.9-42.8%] |
| **>=90** | 1,172/9,140  12.8% [12.1-13.5%] | 879/9,140  9.62% [9.02-10.2%] | 1,761/9,140  19.3% [18.5-20.1%] | 677/9,140  7.41% [6.88-7.97%] | 4,489/9,140  49.1% [48.1-50.1%] |
| **Overall** | 4,377/51,202  8.55% [8.31-8.79%] | 2,950/51,202  5.76% [5.56-5.97%] | 6,457/51,202  12.6% [12.3-12.9%] | 2,509/51,202  4.90% [4.72-5.09%] | 16,293/51,202  31.8% [31.4-32.2%] |

Table S7. Prevalence of cognitive morbidity subtypes by age and sex for the entire acute hospital cohort.

| **Characteristic** | **70-79 years** | | | **80-89 years** | | | **>=90 years** | | |
| --- | --- | --- | --- | --- | --- | --- | --- | --- | --- |
|  | **Female**, N = 9,047*^1^* | **Male**, N = 10,639*^1^* | **p-value**^2^ | **Female**, N = 11,269*^1^* | **Male**, N = 10,673*^1^* | **p-value**^2^ | **Female**, N = 5,542*^1^* | **Male**, N = 3,524*^1^* | **p-value***^2^* |
| Cognitive frailty |  |  | 0.034 |  |  | <0.0001 |  |  | <0.0001 |
| Delirium | 1,049 (12%) | 1,172 (11%) | 1.00 | 1,748 (16%) | 1,502 (14%) | 0.015 | 1,102 (20%) | 688 (20%) | 1.00 |
| Dementia | 423 (4.7%) | 499 (4.7%) | 1.00 | 1,267 (11%) | 1,071 (10%) | 0.020 | 768 (14%) | 379 (11%) | <0.0001 |
| Delirium on dementia | 406 (4.5%) | 393 (3.7%) | 0.028 | 1,398 (12%) | 1,238 (12%) | 0.347 | 952 (17%) | 520 (15%) | 0.013 |
| AMTS<8 | 171 (1.9%) | 194 (1.8%) | 1.00 | 383 (3.4%) | 307 (2.9%) | 0.147 | 270 (4.9%) | 143 (4.1%) | 0.392 |
| None | 6,998 (77%) | 8,381 (79%) | 0.84 | 6,473 (57%) | 6,555 (61%) | <0.0001 | 2,450 (44%) | 1,794 (51%) | <0.0001 |
| 1.. N (%) excludes n=508 for whom sex was not reported  2 Pearson’s chi-squared (for groups overall), and post-hoc testing done using Z-test with Bonferroni correction (for male vs female comparisons) | | | | | | | | | |

Supplementary Figures

Figure S1. Flow chart showing the derivation of the study cohort from ORCHARD-EPR

**Legend: “**Treated in the community” includes patients who received treatment in “hub beds” in care homes and community hospitals for further rehabilitation, recovery and needs assessment outside the acute hospital.

Figure S2. Bespoke EPR cognitive screening proforma as it appears in Cerner Millenium.


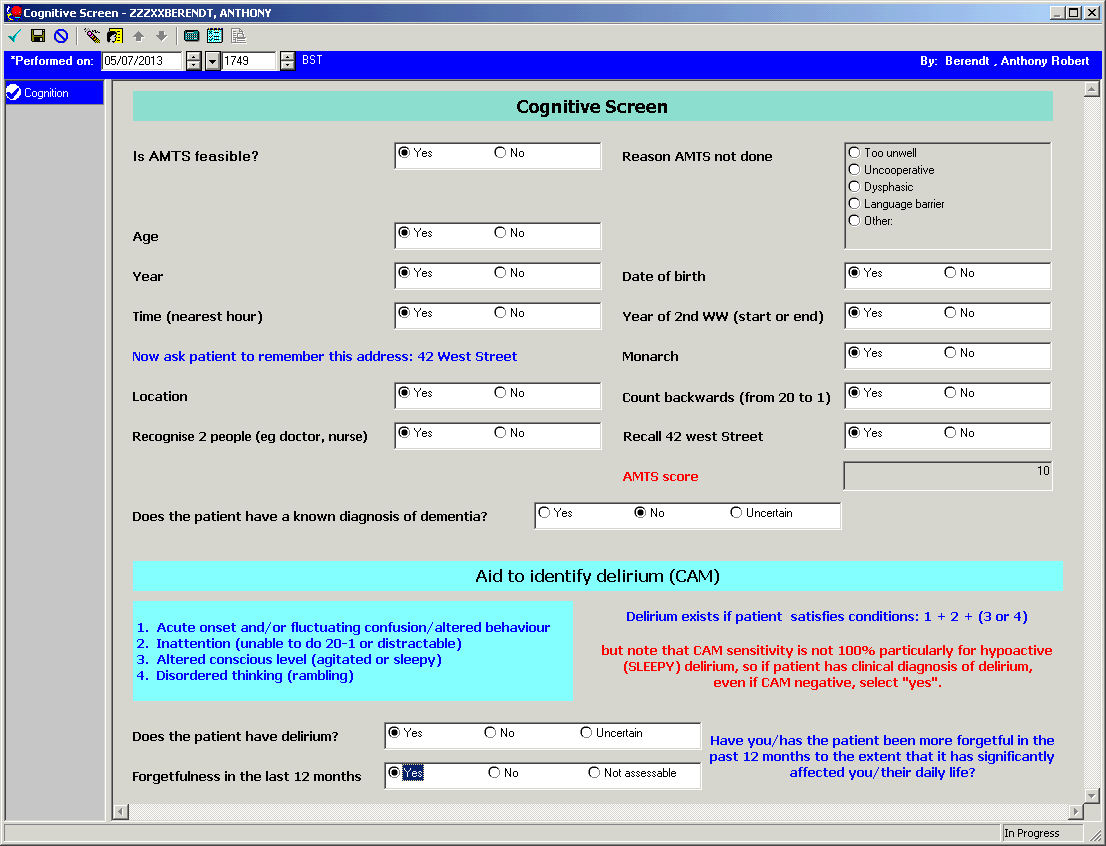


Figure S2 legend. Delirium diagnosis in the cognitive screening proforma was made on the basis of a holistic assessment as part of the clerking process incorporating the AMT, the CAM, observation of the patient, review of notes and interview with informants where appropriate as would be expected to be standard procedure in the assessment of patients with emergency hospital admission.

Figure S3. Source of cognitive morbidity data included in ORCHARD-EPR

**
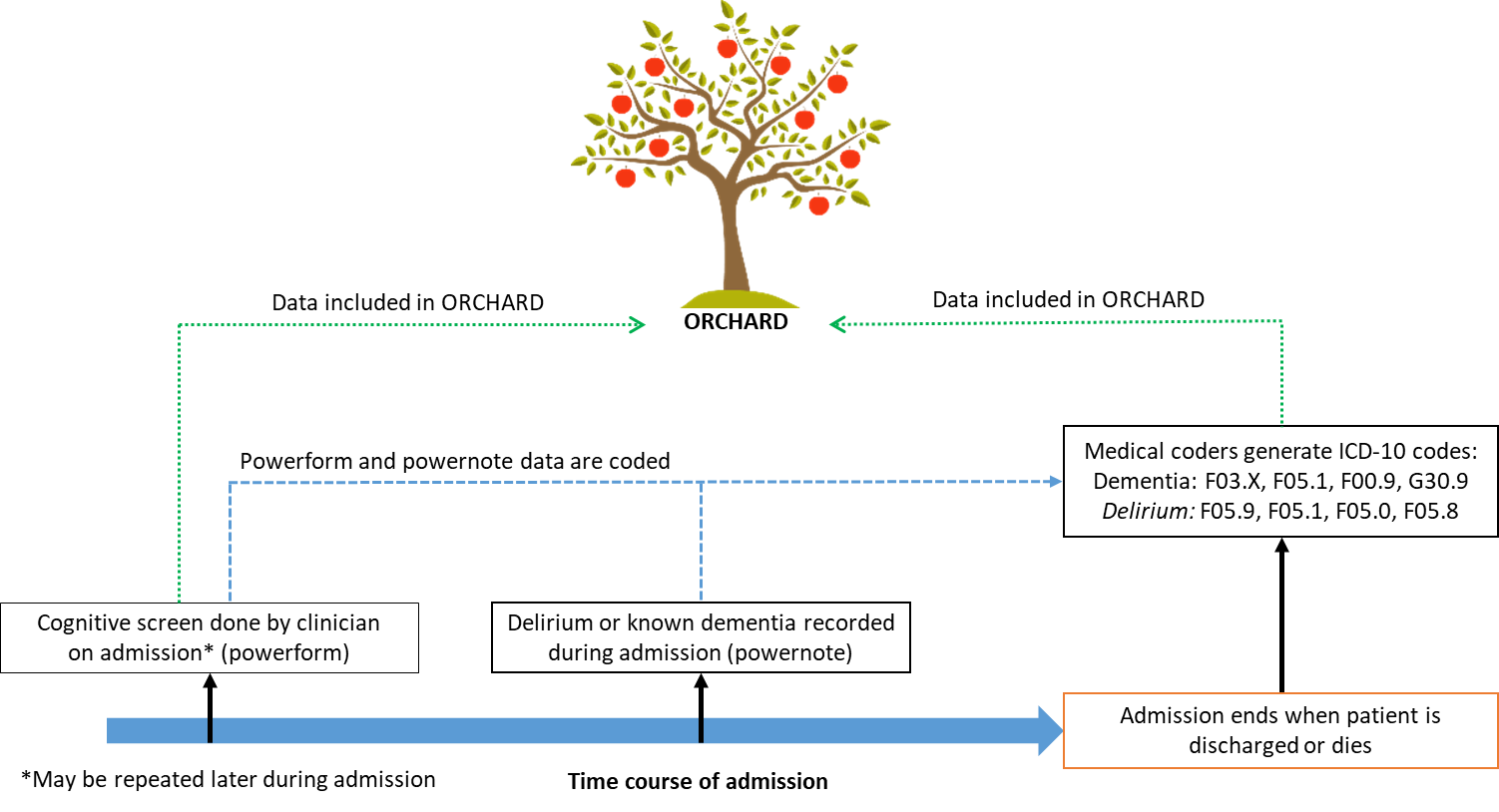
**

Figure S3 legend. ORCHARD-EPR contains only structured data extracted from the EHR (Cerner millenium) and free text entries are not included. Delirium diagnosis in the cognitive screening proforma (powerform), made on the basis of a holistic assessment including the AMT, CAM and review of the notes as part of the clerking process, was extracted from the EHR and uploaded to ORCHARD-EPR. Individual CAM items were not recorded and the CAM was not scored so this information was unavailable. ICD-10 diagnostic codes were also extracted and uploaded to ORCHARD-EPR. ICD-10 codes are allocated after patient discharge by the administrative coding team who review the entire EHR record for that admission as per standard procedures. Therefore, delirium recorded in free text (ie as a powernote) during admission is allocated an ICD-10 code.

For the purposes of the current paper, delirium diagnosis in ORCHARD-EPR was assigned based on either or both of delirium diagnosis in the on-admission cognitive screening proforma or delirium ICD-10 code. Similarly, dementia diagnosis in ORCHARD-EPR was derived from either or both of dementia diagnosis recorded in the on-admission cognitive screening proforma or dementia ICD-10 code. AMT data is also extracted from the EHR and uploaded to ORCHARD-EPR.
